# Intergenerational Hyperglycemia Impairs Mitochondrial Function and Follicular Development and Causes Oxidative Stress in Rat Ovaries Independent of the Consumption of a High-Fat Diet

**DOI:** 10.3390/nu15204407

**Published:** 2023-10-17

**Authors:** Verônyca Gonçalves Paula, Yuri Karen Sinzato, Franciane Quintanilha Gallego, Larissa Lopes Cruz, Ariana Musa de Aquino, Wellerson Rodrigo Scarano, José Eduardo Corrente, Gustavo Tadeu Volpato, Débora Cristina Damasceno

**Affiliations:** 1 Laboratory of Experimental Research on Gynecology and Obstetrics, Gynecology, Postgraduate Course on Tocogynecology, Botucatu Medical School, São Paulo State University (Unesp), Botucatu 18618-687, SP, Brazil; veronyca.paula@unesp.br (V.G.P.); yurisinzato@gmail.com (Y.K.S.); franciane.quintanilha.souza@gmail.com (F.Q.G.); larissa.cruz@unesp.br (L.L.C.); 2 Department of Structural and Functional Biology, Institute of Biosciences, Sao Paulo State University (UNESP), Botucatu 18618-689, SP, Brazil; ariana.musa@unesp.br (A.M.d.A.); wellerson.scarano@unesp.br (W.R.S.); 3 Research Support Office, Botucatu Medical School, São Paulo State University (Unesp), Botucatu 18618-687, SP, Brazil; jecorren@gmail.com; 4 Laboratory of System Physiology and Reproductive Toxicology, Institute of Biological and Health Sciences, Federal University of Mato Grosso (UFMT), Barra do Garças 78600-000, MG, Brazil; gtvolpato@gmail.com

**Keywords:** ovary, mitochondria, high-fat diet, diabetes, rats

## Abstract

We analyzed the influence of maternal hyperglycemia and the post-weaning consumption of a high-fat diet on the mitochondrial function and ovarian development of the adult pups of diabetic rats. Female rats received citrate buffer (Control–C) or Streptozotocin (for diabetes induction–D) on postnatal day 5. These adult rats were mated to obtain female pups (O) from control dams (OC) or from diabetic dams (OD), and they received a standard diet (SD) or high-fat diet (HFD) from weaning to adulthood and were distributed into OC/SD, OC/HFD, OD/SD, and OD/HFD. In adulthood, the OGTT and AUC were performed. These rats were anesthetized and euthanized for sample collection. A high percentage of diabetic rats were found to be in the OD/HFD group (OD/HFD 40% vs. OC/SD 0% *p* < 0.05). Progesterone concentrations were lower in the experimental groups (OC/HFD 0.40 ± 0.04; OD/SD 0.30 ± 0.03; OD/HFD 0.24 ± 0.04 vs. OC/SD 0.45 ± 0.03 *p* < 0.0001). There was a lower expression of MFF (OD/SD 0.34 ± 0.33; OD/HFD 0.29 ± 0.2 vs. OC/SD 1.0 ± 0.41 *p* = 0.0015) and MFN2 in the OD/SD and OD/HFD groups (OD/SD 0.41 ± 0.21; OD/HFD 0.77 ± 0.18 vs. OC/SD 1.0 ± 0.45 *p* = 0.0037). The number of follicles was lower in the OD/SD and OD/HFD groups. A lower staining intensity for SOD and Catalase and higher staining intensity for MDA were found in ovarian cells in the OC/HFD, OD/SD, and OD/HFD groups. Fetal programming was responsible for mitochondrial dysfunction, ovarian reserve loss, and oxidative stress; the association of maternal diabetes with an HFD was responsible for the higher occurrence of diabetes in female adult pups.

## 1. Introduction

The reproductive process of a female depends on the coordinated function of events ranging from the differentiation and release of the mature oocyte to fertilization in order to ensure the successful propagation of the species [1]. The ovary is the central organ in studies on fertility because the ovarian reserve concerns two crucial elements for reproduction: the size of the primordial follicle stock and the quality of the oocytes [2]. In the human species, the formation of the ovarian reserve occurs in embryonic life, and a woman is born with a pool of formed primordial follicles [3]. In rodents, follicular development occurs in postnatal life, and primordial follicles only begin to develop from the third day of birth [4,5]. An apoptosis process controls follicular development, and during each reproductive cycle, follicles undergo apoptosis as a way of being selected in the ovary [6,7].

Ovarian function depends on the combined actions of mitochondrial functions and hormones involved in the reproductive process. In the mitochondria of granulosa cells, energy production occurs, and an environment conducive to the synthesis of lipids and hormones (17 β-estradiol and progesterone) is produced to maintain follicular and oocyte development in the ovary [8,9,10]. Proteins MFF (mitochondrial fission factor) and MFN2 (mitofusin 2) are vital to maintaining mitochondrial dynamics and function by controlling both the fission of mitochondria, which is essential for cell growth and division to maintain an adequate number of mitochondria, and the fusion of mitochondria, which allows for the interconnection of mitochondrial contents and the generation of extensive networks of new mitochondria [11]. The role of these proteins in ovarian development is still not entirely clear. Still, MFF is seen as a regulator of the proliferation and maturation of granulosa cells, and a decrease in its expression is related to an increase in the apoptotic process [12]. MFN2 is a significant target of studies involving reproductive function, and its low expression is known to lead to oxidative stress and impair folliculogenesis [13].

Furthermore, low expressions of MFF and MFN2 are related to problems in embryogenesis. MFN2 knockout mice have been shown to die in utero mid-pregnancy due to placental deficiencies [14,15]. In the presence of mitochondrial dysfunction, ovarian hormone production and increased reactive oxygen species (ROS) may be abnormal, affecting granulosa cell function and oocyte development [16,17].

Women of reproductive age may have diabetes, obesity, and complications resulting from these health problems [18], which leads to impaired follicular development, especially in the presence of hyperglycemia, which may cause infertility [19,20]. Hyperglycemia is associated with mitochondrial dysfunction, oxidative stress, and decreased ovarian reserve [7,21]. There is evidence of alterations in the immunoexpression of markers of cell death by apoptosis, such as a decrease in B-cell lymphoma 2(Bcl-2) and an increase in Bcl-2-associated X protein (Bax), active factor-related apoptosis (FAS), and caspase-3 in the ovaries of diabetic mice, with a consequent increase in apoptosis and a reduction in the number of follicles [7,22]. In addition, hyperglycemia can alter the insulin signaling cascade. This hormone plays a role in ovarian steroidogenesis and follicular development by binding to widespread receptors in the theca and granulosa cells. After binding, intracellular pathways are triggered, including phosphatidylinositol-3-kinase (PI3K) and mitogen-activated protein kinases (MAPK) signaling pathways [23]. PI3K has been explored for its direct participation in ovarian cell proliferation [24,25,26,27], in addition to its activation of the MAPK pathway, including extracellular signal-regulated kinases (ERKs) and p38 kinases. ERK1/2 and p38 effectively participate in ovarian steroidogenesis [28]. These proteins also activate apoptotic mechanisms in granulosa and oocyte cells [29,30,31,32].

Thus, alterations in proteins involved in diabetes-induced insulin resistance, as part of the insulin signaling cascade, may compromise healthy follicular development in the ovaries and interfere with the reproductive process [33,34,35]. These studies are well described in diabetic animals and show increased apoptosis in ovarian follicles, as well as delayed oocyte maturation in mice [19], a decreased pool of follicles with the presence of fibrosis [21], and increased follicular degeneration with increased caspase-3 expression in rats [7].

These repercussions can occur due to intrauterine influences and the postnatal environment, including the consumption of an inadequate diet that also interferes with fetal programming, impairing the reproductive process of the descendants [36,37]. Studies have evaluated the influence of the postnatal diet on reproductive functions. After weaning, rats fed a high-fat diet for 120 days showed insulin resistance, hyperinsulinemia, and altered ovarian morphology [38]. There was a decrease in the levels of ovarian hormones (estrogen and progesterone), an increase in apoptosis in the granulosa cells [39,40], and impairments in the insulin signaling pathway, with a decrease in the expression of insulin receptors in ovarian cells [41,42].

Although there are studies on the repercussions of diabetes and/or a diet with excess fat on the ovaries and the apoptotic mechanisms involved in these animals, there is no investigation on the changes in offspring from an inadequate intrauterine environment caused by maternal diabetes and consume a high-fat diet after weaning. The effect on most rodent organs is similar to the impact on humans. The animal model findings provide a valuable tool to study and better understand the mechanisms involved in metabolic and fetal programming [43].

Given this evidence, considering that a hyperglycemic intrauterine environment or insulin resistance can impair reproductive function both in women and in experimental models, and considering the impossibility of obtaining samples from the ovaries of women of reproductive age, there is a need to study whether changes in animal models persist into the next generation. We hypothesize that female adult pups that develop in a diabetic intrauterine environment and are submitted to a high-fat diet in postnatal life (from weaning to adulthood) will present altered mitochondrial functions and oxidative stress, interfering with follicular development and leading to changes in the ovarian structure and function. These findings may explain lower fertility and impaired reproduction in the adult life of these animals.

## 2. Materials and Methods

### 2.1. Animals

**Figure 1:**
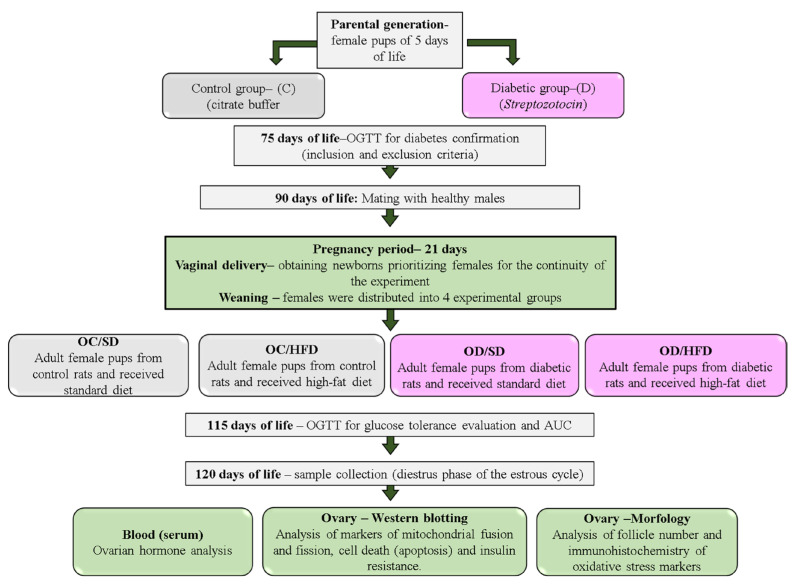
An experimental design of the study.

Female and male Sprague Dawley rats weighing approximately 150 and 250 g, respectively, were adapted and housed at the local laboratory vivarium under standard laboratory conditions (23 ± 2 °C, humidity 50 ± 10%, 12 h light/dark cycle) and fed with a standard diet (Kcal content: 28.54% protein, 62.65% carbohydrate, 8.7% fat, Purina^®^, São Paulo, Brazil). In adulthood (90 days of life), these animals were mated to obtain offspring for diabetes induction or the nondiabetic group (Control). This study was approved by the institution’s Animal Research Ethics Committee (Protocol CEUA Number: 1334/2019) and carried out by the National Institutes of Health Guide for the Care and Use of Laboratory Animals (NIH Publications No. 8023, revised 1978), and all efforts were carried out to minimize animal suffering. A schematic illustration of the experimental design is presented in Figure 1.

### 2.2. Diabetes Induction

For the induction of diabetes in female rats, the beta-cytotoxic drug Streptozotocin (Sigma Aldrich^®^, Burlington, MA, USA—dose of 70 mg/kg, subcutaneously) was used on postnatal day 5 (PND). Glucose levels in the oral glucose tolerance test (OGTT) were classified according to the American Diabetes Association (ADA) [44] and Sinzato et al. [45]. Rats were considered diabetic when, at 75 days of life, they presented glycemia ≥200 mg/dL at least once during OGTT. Female rats not presenting the above characteristics were anesthetized, euthanized, and excluded from this study.

#### Control Group Selection

For the composition of the nondiabetic group (control), the female rats (PND5) received a volume of vehicle (citrate buffer—0.01 M, pH 4.5) equivalent to Streptozotocin as previously described [45]. At 75 days of life, the OGTT was performed and only rats with blood glucose < 140 mg/dL at least three times during the OGTT were included in the control group. Female rats not presenting the above characteristics were anesthetized, euthanized, and excluded from this study. Subsequently, the rats of the control group were mated and went through the pregnancy period; their female pups were used to form the control group that received a standard diet. Previous experiments in our laboratory showed that different generations of control rats did not present changes, but we chose to mate the mothers and use their female pups to mimic the conditions of the diabetic group. All procedures are detailed below.

### 2.3. Mating, Pregnancy, and Lactation

In adulthood, diabetic (D) and control (C) females were mated overnight with nondiabetic normoglycemic male rats. The following morning, pregnancy was confirmed when spermatozoa were found in the vaginal smear, and this was designated as day 0 of pregnancy. Female rats that did not mate after 15 consecutive days were considered infertile and excluded from this study [46]. The offspring were obtained through vaginal delivery, and after birth, at least eight pups per litter were maintained, four males and four females, until weaning (PND 22). When the litter had up to eight pups, there was no manipulation if both sexes were present. When litters exceeded eight pups, males and females were randomly selected. If the litter contained less than eight pups per mother, that mother and her litter were excluded from our experiment but kept in our vivarium for another study.

### 2.4. Experimental Groups and Dietary Patterns 

After weaning, the female adult pups of diabetic (OD) and control (OC) mothers were pseudorandomized by drawing lots, allowing for a maximum of four females per mother, using only two sisters/mother/group. Subsequently, the OD and OC groups were distributed into two other groups according to their diets: standard diet (SD—with Kcal content: 28.54% protein, 62.65% carbohydrate, 8.7% fat, Purina^®^, São Paulo, Brazil) or high-fat diet (HFD—with Kcal content: 23.43% protein, 46.63% carbohydrate, 30% fat, using lard as a fat source).

The rats were distributed into four experimental groups: OC/SD—female adult pups from control mothers and fed a standard diet after weaning; OC/HFD—female adult pups from control mothers and fed a high-fat diet after weaning; OD/SD—female adult pups from diabetic mothers and fed a standard diet after weaning; OD/HFD—female adult pups from diabetic mothers and fed a high-fat diet after weaning. The HFD was handmade at our institution, adequately supplemented with vitamins and minerals, and maintained under refrigeration until the time of use [47]. Given that the diets’ visual characteristics quickly allowed for their distinction, the random housing and blinding of caregivers and/or investigators were impossible.

### 2.5. Oral Glucose Tolerance Test (OGTT) Performance and Area under the Curve (AUC)

In PND 115, OGTT was performed as previously described [48,49] to assess glucose tolerance, and the total glucose response was analyzed by calculating the area under the curve (AUC) using the trapezoidal method [48] and taking into account the amount of glucose circulating in 120 min of OGTT.

### 2.6. Blood and Ovary Collection for Hormonal Analysis 

From the 120th day of life, the rats were analyzed in the vivarium with regard to the phase of the estrous cycle in the morning. The rats that were in diestrus (a stage of the cycle that suffers less hormonal interference) were anesthetized with sodium thiopental (Thiopentax^®^)—dose of 120 mg/kg intraperitoneally) and decapitated. Blood samples were put in test tubes without anticoagulants, incubated on ice for 30 min, and then centrifuged at 1575× *g* for 10 min at 4 °C. The serum was collected and stored at −80 °C until further analysis of hormonal parameters. Subsequently, the rats underwent laparotomy to remove the ovaries. From here, all the procedures were performed with the investigator blinded to the analysis. Serum concentrations of 17 β-estradiol (#501890) and progesterone (#582601) (Cayman Chemical^®^, Ann Arbor, MI, USA) were evaluated by ELISA according to the kit recommendations.

### 2.7. Protein Immunodetection by Western Blotting

One ovary from each rat was removed, immediately frozen in liquid nitrogen, and stored in a freezer at −80 °C. The samples were homogenized in an extraction buffer using RIPA Lysis Buffer (Sigma Aldrich^®^, Burlington, MA, USA) added with protease and a phosphatase inhibitor (Sigma Aldrich^®^, Burlington, MA, USA). Subsequently, they were centrifuged at 1699× *g* at 4 °C for 20 min to remove insoluble material. The determination of the protein concentration was carried out by the Bradford method [50], and the quantification of proteins in the samples was subsequently carried out. Samples were normalized to aliquots containing 70 μg of total protein, and samples (*n* = 6 animals per group) were separated on polyacrylamide gel (DPS-PAGE). The relative molecular weight of the bands was determined according to the color-marked molecular weight standard, Kaleidoscope (Bio-Rad^®^, Hercules, CA, USA), which was also placed in the electrophoretic run. Following electrophoresis, a nitrocellulose membrane was used for protein transfer (Sigma Aldrich^®^, Burlington, MA, USA). Bindings to nonspecific proteins were blocked by incubating the membrane with 5% skimmed milk diluted in a TBS-T buffer for 1 h at room temperature. Membranes were incubated during 16 h at 4 °C with these primary antibodies: MFF (CellSignaling^®^, Danvers, MA, USA #84580, 1:800), Mitofusin-2 (MFN-2) (CellSignaling^®^, Danvers, MA, USA #9482, 1:800), PI3- Kinase (BD Bioscience^®^, Franklin Lakes, NJ, USA #610045, 1:1000), MAPK (Erk1/2) (CellSignaling^®^, Danvers, MA, USA #4695, 1:800), Phospho-p44/42 MAPK (Erk1/2) (CellSignaling^®^, Danvers, MA, USA #4377, 1:800), Bcl-2 (CellSignaling^®^, Danvers, MA, USA #15071, 1:800), FAS (CD-95) (BD Bioscience^®^, Franklin Lakes, NJ, USA #610197, 1:800), Bax (Elabscience^®^ Houston, TX, USA #13814, 1:800), cleaved Caspase-3 (Abcam^®^, Cambridge, UK #ab2302, 1:500), PCNA (PC10) (Santa Cruz^®^, Dallas, TX, USA #sc-56, 1:1000), and β-Actin (CellSignaling^®^, Danvers, MA, USA #3700, 1:1000). After TBS-T washing, the membranes were incubated for 90 min with an HRP-conjugated secondary antibody specific to each primary antibody (CellSignaling^®^, Danvers, MA, USA #7074, #7076, 1:10,000). The reaction was developed by an ECL reagent kit (Bio-Rad^®^, Hercules, CA, USA), and images of the bands were captured using a CCD camera (G: BOX system, Syngene^®^, Cambridge, UK). Target protein bands’ integrated optical densities (IODs) were determined using ImageJ software 1.51s version (National Institutes of Health, Bethesda, MD, USA). Subsequently, expression levels were normalized by the values obtained from the internal control (β-actin), and the results were expressed in fold change with mean ± standard deviation (SD).

### 2.8. Histological Evaluation of the Ovaries

The second ovary of each rat was weighed and fixed in 10% formaldehyde for 24 h, dehydrated in progressive concentrations of alcohol, and embedded in paraffin. Five ovaries/group were used, which were sectioned into sections 8 μm thick, each using a rotating microtome. To perform the conventional histological analysis and count the ovarian follicles, each 10th section was stained with hematoxylin and eosin (H&E) [51]. Images were captured using a computerized imaging system (Software KS-300, version 3.0, Zeiss^®^, Oberkochen BD, German), which received an image from a digital camera (CCD-IRIS/RGB, Sony^®^, Tokyo, Japan), coupled with a microscope (DMR, Leica^®^, Wetzlar HE, German). Counting was performed using Image J^®^ software 1.51s version. Follicles were classified as primordial if each oocyte was surrounded by a single layer of flattened granulosa cells; primary if the oocyte was surrounded by a single layer of cuboidal granulosa cells; growing follicles if they had an oocyte surrounded by at least two layers of cuboidal granulosa cells; and antral follicles if the oocyte contained nuclear material to avoid the double counting of more significant follicle types that could span multiple sections [51]. This structure was analyzed in a single image to ensure no corpus luteum was counted twice (magnification 4×). It was followed through the ovary and counted only once, even though it appeared in the next section to be counted [51].

### 2.9. Immunohistochemical (IHC) Evaluation of the Ovaries

For the IHC technique, every 9th section was considered, and corpus luteum cells, stromal cells, and granulosa cells were the analyzed structures. Primary antibodies Superoxide dismutase-1 (anti-SOD-1, Abcam^®^ Cambridge, UK #ab16831, 1:200), Catalase (anti-catalase, Abcam^®^ Cambridge, UK #ab52477, 1:50), and Malondialdehyde (anti-MDA, Abcam^®^ #ab6463, 1:500) were used. There was no antigenic recovery. Endogenous peroxidase blockade was performed with methanol and hydrogen peroxide (at a concentration of 20%) for 40 min at room temperature and with the use of a ready-to-use blockade (peroxidase inhibitor containing hydrogen peroxide and 15 mM sodium azide—Dako^®^, Santa Clara, CA, USA), with incubation in an oven at 27 °C for 20 min. For blocking nonspecific proteins, skimmed milk blocks at a concentration of 10% were used for 2 h, and Protein Block (casein 0.25% in PBS, containing carrier protein and sodium azide 15 mM—Dako^®^, Santa Clara, CA, USA) was used for 30 min in an oven at 27 °C. After primary antibody incubation, the secondary antibody (Histofine^®^, Nichirei Bio, Tokyo, Japan) was added for 30 min in an oven at 27 °C. DAB chromogen (3,3 diaminobenzidine) was used for 3 min at room temperature for peroxidase development. Then, the slides were counterstained in Harris hematoxylin and mounted. Images were captured using a computerized imaging system (Software KS-300, version 3.0, Zeiss^®^, Oberkochen BD, German), which received an image from a digital camera (CCD-IRIS/RGB, Sony^®^, Japan), coupled with a microscope (DMR, Leica^®^). Both antibodies showed cytoplasmic immunostaining, and analyses were performed by immunostaining intensity using Image J^®^ software 1.51s version.

### 2.10. Statistical Analysis

#### 2.10.1. Sample Size Calculation

Sample size calculation was performed by taking into account the mean and standard deviation values previously obtained from the Area Under Curve (AUC) and using the SAS—STATISTICAL ANALYSIS SYSTEM Software version 9.4, 2021. Based on the AUC values, using 90% power and 5% type I error, the effect size was determined by a specialist in Biostatistics at our institution, with a minimum of 5 rats per group of female pups. For immunohistochemical analysis, calculation was estimated in five photos/structure/animal/group adapted from previous results for the analysis of SOD intensity in other tissues using the same software.

#### 2.10.2. Statistical Tests

Data are presented as mean ± standard deviation. For the AUC and serum concentrations of progesterone and 17β-estradiol, Two-way ANOVA followed by Tukey’s Multiple Comparison Test was used. For data that did not show normal distribution (Gauss curve), adjustments were made to all data and then analyzed by distribution tests, including the protein expression of different markers and marking intensity for SOD-1, Catalase, and MDA, which were submitted to Gamma Distribution tests, followed by Wald’s Multiple Comparisons Test. To analyze the count of primordial, primary, and antral follicles, as well as corpora lutea, the Poisson distribution test was used followed by the Wald test, as in these cases, the mean and variance values were close. To analyze the count of growing follicles, the negative Binomial test was followed by the Wald test, as there was an overdispersion of variance and mean values. Pearson’s correlation test was used to analyze the existence of any potential linear association between quantitative data. For data analysis, the statistical SAS—STATISTICAL ANALYSIS SYSTEM Software version 9.4, 2021 was used, and a *p*-value of <0.05 was regarded as statistically significant.

## 3. Results

### 3.1. Intrauterine Exposure to Maternal Hyperglycemia Increases the Percentage of Female Adult Pups with Diabetes

The area under the curve (AUC) and percentage (%) data of female adult pups that developed diabetes at 115 days of life (PND 115) are shown in Table 1. The amount of circulating glucose during the 120 min of OGTT was higher in the OC/HFD, OD/SD, and OD/HFD groups compared to the control group (OC/SD). Female adult pups of diabetic rats who consumed a high-fat diet in postnatal life (OD/HFD) had an even higher AUC compared to the OC/HFD and OD/SD groups, leading to a higher percentage of rats that became diabetic in the OD/HFD group (40.00%) in relation to the other groups (11.11%).

### 3.2. Changes in Progesterone Concentrations and the Estrous Cycle of Female Adult Pups Are Influenced by Maternal Hyperglycemia and Postnatal HFD Consumption

**Figure 2:**
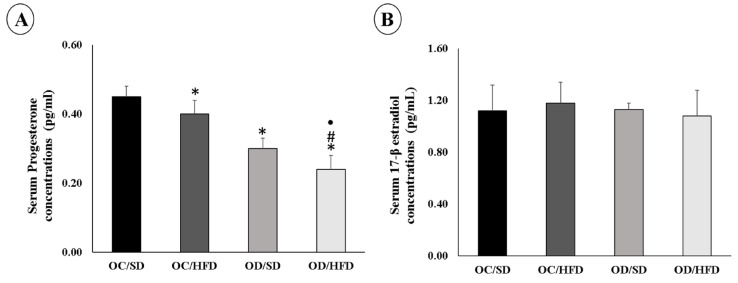
The serum concentrations of (**A**) Progesterone and (**B**) 17 β-estradiol of female adult pups from the control (OC) or diabetic (OD) groups that consumed a standard (SD) or high-fat diet (HFD) from weaning up to 120 days of postnatal life. Values were expressed as mean ± standard deviation. *n* = 8 rats/group; * *p* < 0.05—compared to the OC/SD group; # *p* < 0.05—compared to the OC/HFD group; • *p* < 0.05—compared to the OD/SD group (ANOVA followed by Tukey’s Multiple Comparison Test).

At 120 days of life of the female adult pups, there was a decrease in serum progesterone concentrations in the OC/HFD, OD/SD, and OD/HFD groups compared with the OC/SD group (Figure 2A). Progesterone concentrations were lower in the OD/HFD group than in the OC/HFD and OD/SD groups. On the other hand, serum concentrations of 17β-estradiol did not differ between groups (Figure 2B). Estrous cycle assessments were performed in both groups of the female adult pups of diabetic rats, and the OD/SD group remained longer in the estrus phase (*p* = 0.0332).

### 3.3. Exposure to Maternal Hyperglycemia Alters the Expression of Proteins Involved in Mitochondrial Function, Apoptosis, and Cell Proliferation in the Ovaries of Female Adult Pups

**Figure 3:**
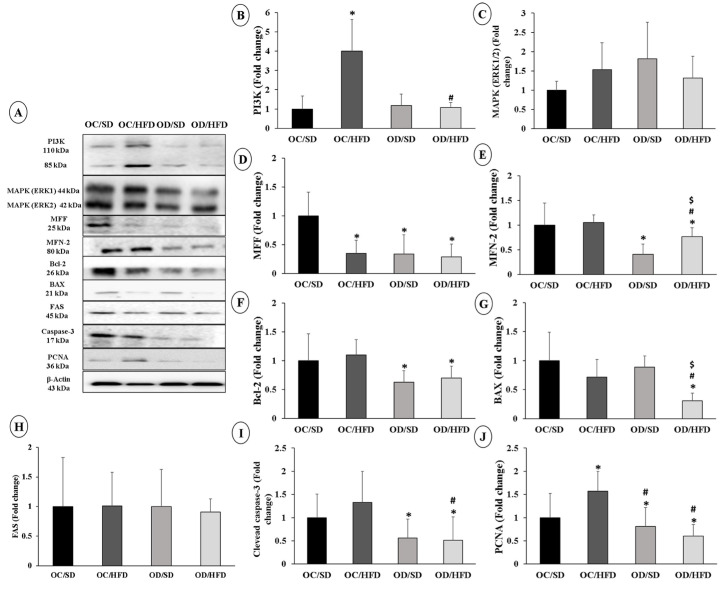
Protein expression analysis involved in the insulin signaling pathway, mitochondrial dynamics, and apoptosis. (**A**) Membranes, (**B**) PI3K, (**C**) MAPK (ERK1/2), (**D**) MFF, (**E**) MFN-2, (**F**) Bcl-2, (**G**) Bax, (**H**) FAS, (**I**) Caspase-3 cleaved and (**J**) PCNA of female adult pups from control (OC) or diabetic (OD) groups that consumed a standard (SD) or high-fat diet (HFD) from weaning up to 120 days of postnatal life. Values were expressed as mean ± standard deviation. *n* = 6 rats/group; * *p* < 0.05—compared to the OC/SD group; # *p* < 0.05—compared to the OC/HFD group; $ *p* < 0.05—compared to the OD/SD group (Gamma Distribution test and ANOVA followed by Wald’s multiple comparisons test).

The expression of proteins related with the insulin signaling pathway was evaluated. PI3K protein expression was higher in the OC/HFD group compared to the OC/SD and OD/HFD groups (Figure 3B). Regarding MAPK expression (ERK1/2—phosphorylated and total), there were no changes among the groups (Figure 3C).

The analysis of the expression of proteins involved in mitochondrial function showed a decrease in the expression of the MFF protein in the OC/HFD, DF/DP, and DF/DHL groups when compared with the expressions of the FC/DP rats (Figure 3D). The MFN-2 protein was less expressed in the groups of the daughters of diabetic rats compared to the control group (OC/SD). In the OD/HFD group, MFN-2 expression was also lower than in the OC/HFD OD/SD groups (Figure 3E).

Markers related to the process of apoptosis and cell proliferation were also altered. There was a decrease in Bcl-2 expression in the diabetic groups (OD/SD and OD/HFD) compared with the control group (OC/SD) (Figure 3F). Bax expression was also lower in the OD/HFD group than in other groups (Figure 3G). The female adult pups of diabetic rats showed a decrease in the expression of cleaved Caspase-3 (Figure 3I) and of the cell proliferation marker PCNA compared to the OC/SD and OC/HFD groups (Figure 3J). There were no changes in FAS expression between groups (Figure 3H).

### 3.4. Maternal Hyperglycemia Impairs Ovarian Follicular Development in Female Adult Pups 

The number of ovarian follicles is shown in Table 2. There was a decrease in primordial, primary, and growing follicles in the OD/SD and OD/HFD groups compared with the OC/SD group. The OC/HFD, OD/SD, and OD/HFD groups showed a decrease in antral follicles when compared with the OC/SD group; in the OD/HFD group, there was also a decrease in antral follicles compared with the OC/SD group. There were no changes in the corpus luteum number.

### 3.5. Maternal Hyperglycemia Associated with Postnatal HFD Consumption Leads to Oxidative Stress in the Ovarian Cells of Female Adult Pups

**Figure 4:**
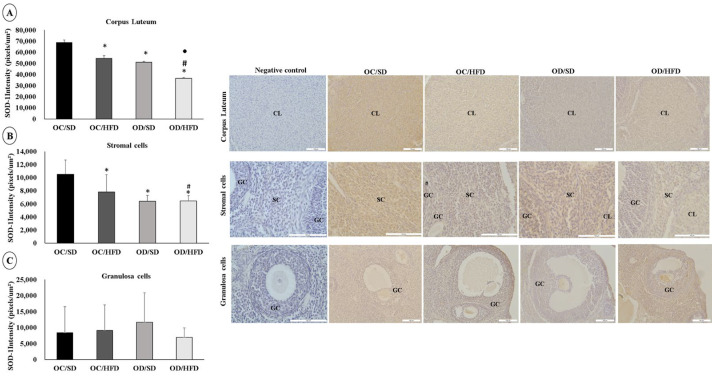
Analysis of the labeling intensity of superoxide dismutase (SOD-1). (**A**) corpus luteum, (**B**) stromal cells, and (**C**) granulosa cells of female adult pups from control (OC) or diabetic (OD) groups that consumed a standard (SD) or high-fat (HFD) diet from weaning up to 120 days of postnatal life. Values were expressed as mean ± standard deviation. *n* = 5 rats/group; Legends: CL: corpus luteum; SC: stromal cells; GC: granulosa cells; Magnification: 20× for corpus luteum and granulosa cells; 40× for stromal cells; * *p* < 0.05—compared to the OC/SD group; # *p* < 0.05—compared to the OC/HFD group; • *p* < 0.05—compared to the OD/SD group (Gamma Distribution test and ANOVA followed by Wald’s multiple comparisons test).

**Figure 5:**
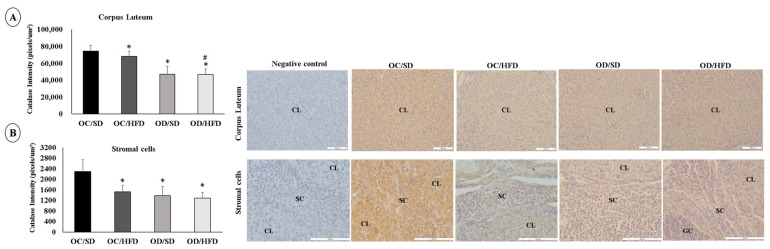
Analysis of the labeling intensity of Catalase. (**A**) corpus luteum, (**B**) stromal cells of female adult pups from control (OC) or diabetic (OD) groups that consumed a standard (SD) or high-fat diet (HFD) from weaning up to 120 days of postnatal life. Values were expressed as mean ± standard deviation. *n* = 5 rats/group; Legends: CL: corpus luteum; SC: stromal cells; GC: granulosa cells; Magnification: 20× for corpus luteum and 40× for stromal cells; * *p* < 0.05—compared to the OC/SD group; # *p* < 0.05—compared to the OC/HFD group (Gamma Distribution test and ANOVA followed by Wald’s multiple comparisons test).

The staining intensity for SOD-1 and Catalase was lower in corpus luteum cells in the OC/HFD, OD/SD, and OD/HFD groups compared with the OC/SD group. The OD/HFD group also showed a lower staining intensity for SOD-1 compared with the other groups (OC/SD, OC/HFD, and OD/SD) and lower staining intensity for Catalase compared with the OC/HFD group (Figure 4A and Figure 5A). In stromal cells, the staining intensity for SOD-1 and Catalase was also lower in the OC/HFD, OD/SD, and OD/HFD groups compared with the OC/SD group and even lower in the OD/HFD group for SOD-1 compared with the OC/HFD group (Figure 4B and Figure 5B). In granulosa cells, there was an increase in staining intensity for SOD-1 in the OD/SD group compared to the OC/SD and OD/HFD groups (Figure 4C). Regarding the intensity of Catalase staining in granulosa cells, microscopic analysis was complicated due to the need for different magnification lenses due to the high variability in follicle sizes. Therefore, these data were not presented. However, there was no difference in the intensity of Catalase staining in granulosa cells between groups (*p* = 0.442).

**Figure 6:**
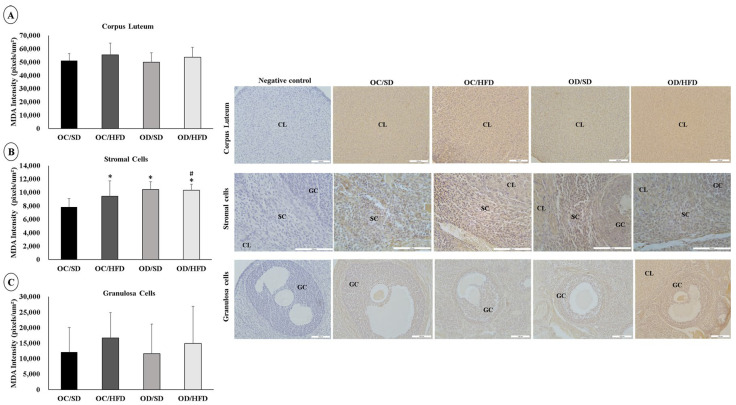
Analysis of the labeling intensity of Malondialdehyde (MDA). (**A**) corpus luteum, (**B**) stromal cells, and (**C**) granulosa cells of female adult pups from control (OC) or diabetic (OD) groups that consumed a standard (SD) or high-fat diet (HFD) from weaning up to 120 days of postnatal life. Values were expressed as mean ± standard deviation. *n* = 5 rats/group; Legends: CL: corpus luteum; SC: stromal cells; GC: granulosa cells; Magnification: 20× for corpus luteum and granulosa cells; 40× for stromal cells; * *p* < 0.05—compared to the OC/SD group; # *p* < 0.05—compared to the OC/HFD group (Gamma Distribution test and ANOVA followed by Wald’s multiple comparisons test).

There were no changes in the intensity of MDA labeling in corpus luteum cells between groups (Figure 6A). There was an increase in staining intensity for MDA in the stromal cells of the OC/HFD, OD/SD, and OD/HFD groups compared with the OC/SD group (Figure 6B). The staining intensity for MDA in the OD/DHL group was even higher compared to the OC/HFD group (Figure 6B). There was a higher staining intensity for MDA in granulosa cells in the OC/HFD and OD/HFD groups compared with the OC/SD rats (Figure 6C).

### 3.6. Correlations between the Data of AUC, Serum Progesterone Concentration, Markers of Apoptosis, Mitochondrial Function, and Oxidative Stress in Female Pups

**Figure 7:**
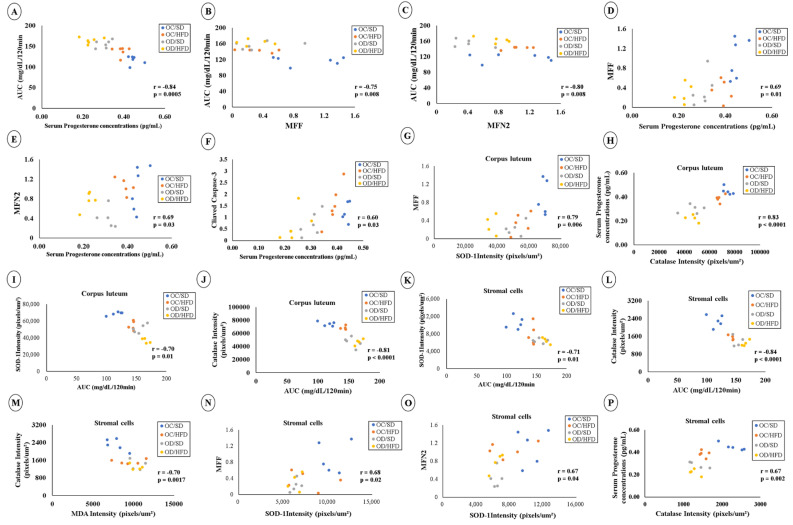
Correlation analysis between data. (**A**) Correlation between AUC/120 min × serum progesterone; (**B**) Correlation between AUC/120 min × MFF; (**C**) Correlation between AUC/120 min × MFN2; (**D**) MFF × Serum progesterone concentrations; (**E**) MFN2 × serum progesterone concentrations; (**F**) Correlation between cleaved Caspase-3 × serum progesterone concentrations; (**G**) MFF × SOD−1 intensity in corpus luteum; (**H**) CAT intensity × serum progesterone concentrations in corpus luteum; (**I**) AUC/120 min x SOD−1 intensity in corpus luteum; (**J**) AUC/120 min × CAT intensity in corpus luteum; (**K**) SOD-1 intensity × AUC/120 min in stromal cells; (**L**) CAT intensity × AUC/120 min in stromal cells; (**M**) CAT intensity × MDA intensity in stromal cells; (**N**) MFF × SOD−1 intensity in stromal cells; (**O**) MFN2 × SOD−1 intensity in stromal cells and (**P**) correlation between CAT intensity × serum progesterone in stromal cells. *p* < 0.05—Pearson correlation test.

Data correlation analyzes are shown in Figure 7. Regarding blood glucose, there were negative correlations between AUC/120 min × serum progesterone (Figure 7A), AUC/120 min × MFF (Figure 7B), and AUC/120 min × MFN2 (Figure 7C). Positive correlations were found between MFF × serum progesterone (Figure 7D), MFN2 × serum progesterone (Figure 7E) and cleaved Caspase-3 × serum progesterone (Figure 7F). Among corpus luteum cells, there was a positive correlation between MFF × SOD-1 intensity (Figure 7G) and CAT intensity × serum progesterone (Figure 7H); there were negative correlations between AUC/120 min × SOD-1 intensity (Figure 7I) and AUC/120 min × CAT intensity (Figure 7J). In ovarian stromal cells, there were negative correlations between SOD-1 intensity × AUC/120 min (Figure 7K), CAT intensity × AUC/120 min (Figure 7L), and CAT intensity × MDA intensity (Figure 7M), in addition to positive correlations between MFF × SOD-1 intensity (Figure 7N), MFN2 × SOD-1 intensity (Figure 7O), and CAT intensity × serum progesterone (Figure 7P) in stromal cells.

## 4. Discussion

The relationship between diabetes and ovaries, as well as HFD consumption, is already established, but the intergenerational consequences are still poorly researched [7,19,40,52,53]. In this study, we evaluated the intergenerational effect of hyperglycemia on the ovaries of female adult pups of diabetic rats. In addition, we also assessed how the postnatal environment (HFD consumption) might negatively influence or exacerbate ovarian repercussions. Our study focused on the repercussions for the offspring after exposure to maternal hyperglycemia and the consumption of an HFD, although there is evidence that other exposure factors, such as environmental, epigenetic, and genetic factors of paternal origin, can also compromise the adequate development of the individual and their quality of life and longevity [54]. This is the first study to investigate how these two factors (diabetes and HFD) interfere with hormone secretion, mitochondrial function, ovarian reserve, and oxidative stress in the ovaries.

We demonstrated that maternal hyperglycemia and postnatal HFD consumption compromised the glycemic metabolism of female adult pups. When analyzing the OGTT, we observed greater circulating glucose in the OC/HFD, OD/SD, and OD/HFD groups. The female adult pups of diabetic rats developed diabetes, and when associated with HFD consumption, the proportion of diabetic rats was even higher.

Previously, in our laboratory, it was verified that female adult pups of diabetic rats, whether or not they consumed an HFD from weaning to adulthood, showed a decrease in serum insulin concentration, in addition to an increase in insulin-marked cells in the pancreas, suggesting that the secretion of insulin and not its signaling would be compromised in these rats [47]. Therefore, to analyze the influence of the association of diabetes and HFDs on markers of insulin resistance, the expression of transcriptional proteins related to the insulin signaling pathways PI3K/Akt and MAPK (ERK1/2) were evaluated in ovarian homogenates.

The OC/HFD group showed an increased expression of PI3K. It has already been demonstrated that the PI3K/Akt and MAPK (ERK1/2) pathways promote glucose transport, cell proliferation, and cell differentiation; follicular apoptosis interacts with gonadotropins to modulate ovarian hormone secretion [55,56,57], in addition to being related to mitochondrial function in the ovary [58]. The greater expression of PI3K in the control group that consumed an HFD may indicate the increased activation of this pathway. In contrast, the opposite can be seen in the female adult pups of diabetic mothers who consumed an HFD. The PI3K pathway is related to steroidogenesis [59]. The female adult pups of diabetic mothers showed decreased serum progesterone concentrations and changes in the proteins responsible for mitochondrial fission and fusion.

According to our findings, the female adult pups of diabetic rats showed changes in proteins responsible for mitochondrial fission and fusion. Studies on the relationship between proteins responsible for mitochondrial function and ovaries are scarce. Furthermore, no investigations of the expression of MFN2 or MFF in the ovaries of female adult pups of diabetic rats were found. The causal relationship between diabetes and mitochondrial abnormalities is evidenced in studies that show that there is dysfunction in mitochondria in β-cell stimulus–secretion coupling, leading to a reduction in ATP generation by mitochondria and β-cell dysfunction [60]. Furthermore, the morphology of mitochondria in type 2 diabetes is altered with very small or very large mitochondria, reduced mitochondrial activity, and the high generation of ROS, which characterize the pancreatic dysfunction of this disease [61,62], in addition to the repercussions on skeletal muscles, which are also widely reported [63]. According to the DOHaD concept, these mitochondria already altered by diabetes can then be transmitted to offspring, and interactions with nutritional and metabolic insults may reflect the changes found in our model.

The literature shows that both diabetes and an HFD can impair MFF and MFN2 expression in other tissues, such as cardiac tissue in male rats [64] and skeletal muscles in diabetic patients [65]. In our laboratory, it was verified that female adult pups of diabetic rats at 150 days of life also showed a decrease in the expression of MFN2 and MFF in the skeletal muscles (unpublished data).

It is interesting to note that although both groups of the female pups of diabetic mothers showed a larger MFN2 expression than the control group, the expression of this protein was higher in the OD/HFD group than in the OD/SD group. It has been shown that diabetes during pregnancy leads to mitochondrial dysfunction, and these defective mitochondria can be transmitted to the developing offspring [66,67]. HFD consumption presents controversial results; some studies observed the overstimulation of mitochondrial functions in the skeletal muscle of rodents [68,69], while others showed a lower mitochondrial function when animals consumed an HFD [70,71]. Despite the lack of studies on MFN2 in the ovaries, it has been verified that short-term HFD consumption increased MFN2 expression in the skeletal muscle of young rats, suggesting an adaptation in mitochondrial metabolism that precedes the appearance of metabolic disorders late in the lives of these animals [72]. In our model, HFD consumption by the female pups of diabetic mothers may have led to this mechanism to try to compensate for maternal hyperglycemia-programmed mitochondrial dysfunction. However, it was ineffective because neither mitochondrial function nor other parameters were improved or analyzed. Thus, these findings must be further investigated.

MFN2 is essential in maintaining mitochondrial DNA integrity, and its abnormality can affect granulosa cell function and oocyte development [13]. In vitro studies using cell lines with an MFN2 blockade [73] and also in vivo, using a model where mice treated with cisplatin had low MFN2 expression in the ovaries [13,74], were associated with the decreased production of steroid hormones [74]. Thus, we suggest that hyperglycemia was responsible for the low expression of MFN2 and MFF and, consequently, led to changes in follicular cells that compromised the synthesis of ovarian progesterone, confirmed by the low concentration of serum progesterone in the female adult pups of diabetic rats, regardless of consumption of an HFD. Our correlation analysis confirmed that the increase in the glucose concentration in the OGTT impacted the decrease in the expression of MFN2 and MFF and in the concentration of progesterone. In our model, the correlations show that the reduction in progesterone seems to be associated with mitochondrial dysfunction. This result reflects losses in the reproduction of these rats, as it is an essential hormone for the maintenance of pregnancy [75]. In fact, in another study with the same model, there was an increase in embryo losses before implantation in the female adult pups of diabetic mothers, whether consuming HFDs or not, and a reduction in the implantation and live fetus numbers in the female adult pups of diabetic mothers who consumed an HFD [76].

Although our results do not show differences in 17β-estradiol concentrations, there was a more extended stay in the estrus phase of the estrous cycle in the OD/SD group. According to the literature, a more extended stay in the estrus phase is often accompanied by increased estradiol concentrations and a greater recruitment of follicles to develop [77,78]. A possible justification for our finding is that the 17β-estradiol dosage that occurred with the animals in the diestrus stage of the estrous cycle was characterized by a decrease in the concentrations of this hormone precisely because it is a progestogenic phase [79]. This point can be considered a limitation of our study.

The survival of ovarian follicles depends on several signaling factors that regulate cell proliferation and death (apoptosis) [80]. Apoptosis is a complex process involving intrinsic and extrinsic activation pathways by pro- and anti-apoptotic proteins coordinated by normal mitochondrial function [81]. Studies have demonstrated that the intrinsic pathway of apoptosis controlled by the Bcl-2 family has an important role in oocyte survival and in the maintenance of the ovarian reserve [82]; changes in this pathway, such as the increased activation of pro-apoptotic members (e.g., Bax) and a decrease in anti-apoptotic members (e.g., Bcl-2) compromise reproduction through caspase-3-mediated cell death [22,83]. Furthermore, impaired cell proliferation prevents adequate follicular development and oocyte maturation, leading to reproductive dysfunction [84]. It is well established that diabetes leads to disorders in the apoptotic and cell proliferation pathways and affects several tissues, including the ovary.

The relationship between hyperglycemia and the induction of apoptosis has already been established, and studies have reported the presence of excess apoptosis in the ovarian cells of diabetic rats [22,83,85] and in the ovaries of rats that consumed an HFD by increasing the expression of Bax and caspase-3 in addition to other apoptotic markers [18,52,86,87]. However, our results are controversial. We could verify that Bcl-2 and Caspase-3 expression in the ovaries was lower in the female adult pups of diabetic rats, and the OD/HFD group showed even lower Bax expression. The fact that the female adult pups of diabetic rats exposed to an HFD showed a decrease in pro and anti-apoptotic markers may be related to the possible impairment of cell proliferation because the expression of PCNA, which acts in the synthesis, repair, and replication of DNA [88], was lower in the female adult pups of diabetic rats.

In other models, it has already been shown that mitochondrial dysfunction leads to decreased PCNA expression and cell proliferation damage [89]. In addition, the low expression of MFF impairs cell proliferation by hindering their nuclei’s fission and the process of division by mitosis [16]. Our observations are unprecedented in the literature, and no data are available about the mechanism of action of PCNA on mitochondrial function in the ovaries. However, recently, PCNA was recognized as an important marker of folliculogenesis, as it is present in a greater concentration in the granulosa cells of secondary and antral follicles [90], which justifies the relationship between the low protein expression of PCNA, failures in follicular cell proliferation, and reduction in progesterone synthesis in our study. Lower levels of caspase-3 and PCNA expression in the female adult pups of diabetic rats, regardless of HFD consumption, may indicate that even the apoptosis that should occur physiologically does not seem to happen, which could be a cause or consequence of the low proliferation of cells in the ovaries. In a study using a model of severe diabetes induced by alloxan, a direct relationship was established between hyperglycemia and low PCNA expression in rat myometrial cells, with hormonal dysfunction caused by hyperglycemia being responsible for intense cell proliferation [91]. A positive correlation was found between the low expression of caspase-3 cleaved in the ovaries and the low concentration of progesterone, and it has already been demonstrated that the production of progesterone seems to be dependent on the increase in the activation of caspase-3 in the granulosa cells of pre-ovulatory follicles [92]. Furthermore, the loss of proliferative capacity may be associated with other pathways, such as cellular senescence and autophagy [93]. Sun et al. [94] demonstrated that oxidative stress in mouse ovarian cells decreases hormonal production and cell proliferation, accelerating granulosa cells’ senescence through excessive autophagy activation. We did not assess senescence or autophagy markers in our model, and we consider this as limitation of our study. The hyperglycemia-induced oxidative stress in ovarian cells might indicate that low cell proliferation and apoptosis is related to early ovarian senescence in the female adult pups of diabetic mothers.

Interestingly, the mechanism involved in responses to HFD consumption by the control rat group seems to follow another pattern. In the OC/HFD group, there was a greater expression of PCNA, indicating an increase in cell proliferation in this group, which may be a consequence of the increased activation of the PI3K pathway. Increased cell proliferation can result in greater follicular activation, which recruits more follicles to develop, but harms reproductive function as more follicles are lost [86,95]. More significant proliferation and follicular loss were observed in rats fed HFDs, indicating decreased ovarian reserve [52,96]. However, this was not verified in our study. The analysis of the ovarian follicle count showed that the OC/HFD group rats did not present changes in the ovarian reserve or the growing follicle count. However, a decrease in the antral follicle count was verified. The greater activation of the PI3K pathway and the increase in cell proliferation in this group may have occurred as a compensatory mechanism to prevent follicular loss, which did not happen in the female adult pups of diabetic rats.

Follicular development was impaired in the female adult pups of diabetic rats regardless of HFD consumption. The decrease in the primordial follicle count shows that there was a loss of ovarian reserve. This result shows a lower count of growing follicles and antral follicles. This result corroborates the literature, as diabetes leads to a decrease in ovarian reserve both in experimental models and in clinical studies [7,21,97,98] and shows that the harmful effect is transmitted between generations. The low expression of MFF and MFN2 can decrease the reserve by hindering the fission and fusion of cell nuclei and impairing the proliferation of granulosa cells [99]. MFN2 knockout mice drastically decreased in primordial and growing follicles at 12 months of life, leading to ovarian failure [58]. Thus, the loss of ovarian reserve in our model may be a direct consequence of mitochondrial dysfunction and damage to the process of apoptosis and cell proliferation caused by hyperglycemia. Furthermore, this finding is also related to increased oxidative stress in ovarian cells.

In our study, the immunohistochemical analysis of the ovary showed that the rats in the control group that consumed an HFD (OC/HFD) and both groups of the female adult pups of diabetic mothers (OD/SD and OD/HFD) showed a decrease in the intensity of enzyme marking antioxidants SOD-1 and Catalase in stromal cells and corpus luteum and increased marking of lipid peroxidation by MDA in stromal cells. Both intrauterine exposure to maternal diabetes and HFD consumption led to oxidative stress in ovarian cells. In the ovary, the presence of reactive oxygen species (ROS) is required for follicle rupture and ovulation. However, there needs to be a balance between ROS and antioxidant agents, as oxidative stress accelerates the loss of follicles [17] and, consequently, impairs the secretion of ovarian hormones [100]. Hyperglycemia is associated with the high production of ROS by mitochondria [101], and oxidative stress is one of the hallmarks in experimental models of diabetes [102] and/or HFDs [103,104].

Our findings on the low intensity of SOD-1 and Catalase labeling in stromal cells are interesting data, as there is little information on the role of the stroma in folliculogenesis and the ovarian cycle. Kinnear et al. [105] showed that stromal cells have subpopulations that play different roles in maintaining ovarian dynamics, mainly because follicular and luteal cells are formed from them. In addition, a specific subpopulation called theca interstitial cells (which later migrate to become theca cells themselves) have been recognized for their ability to synthesize hormones, as well as the presence of hormone receptors [106,107]. However, it is still necessary to clarify the role of the stroma in hormone production and the mechanisms by which these cells influence folliculogenesis [105].

Although our data bring valuable information about the association between diabetes and HFDs on ovarian dynamics, some limitations can be pointed out, such as the presence of biometric parameters for evaluating a possible catch-up growth in the offspring of diabetic mothers and the evaluation of other proteins involved in mitochondrial dynamics such as Drp1, MFN1, and OPA1, which could not be evaluated in this study. Independent of the limitations, our study is the first to provide information about mitochondrial function in rats born to diabetic mothers, and our data bring a new perspective on how hyperglycemia interferes with future generations.

## 5. Conclusions

In conclusion, mitochondrial dysfunction caused by hyperglycemia was a determinant for the alterations found in our model. The negative repercussions indicate that the female adult pups of diabetic rats, regardless of HFD consumption, have mitochondrial dysfunction with impaired proliferation, hormone secretion, and oxidative stress, as well as decreased ovarian reserve. Thus, our findings indicate a possible model for studying ovarian dynamics and fertility.

## Figures and Tables

**Table 1:** Area under the curve (AUC) and percentage (%) of rats with diabetes (female adult pups) from the control (OC) or diabetic (OD) groups that consumed a standard (SD) or high-fat diet (HFD) from weaning up to 120 days of postnatal life.

Groups	AUC (mg/dL/120 min)	% Rats with Diabetes
OC/SD	117.30 ± 10.37	0%
OC/HFD	138.68 ± 11.01 *	0%
OD/SD	155.97 ± 9.35 *	11.11%
OD/HFD	164.70 ± 7.24 *^#$^	40.00% *^#$^

Values were expressed as mean ± standard deviation. *n* = 8 rats/group; * *p* < 0.05—compared to the OC/SD group; ^#^ *p* < 0.05—compared to the OC/HFD group; ^$^ *p* < 0.05—compared to the OD/SD group (ANOVA followed by Tukey’s Multiple Comparison Test).

**Table 2:** The ovarian follicle count of female adult pups from the control (OC) or diabetic (OD) groups that consumed a standard (SD) or high-fat diet (HFD) from weaning up to 120 days of postnatal life.

Variables/Groups	SD	HFD
Primordial Follicles		
OC	75.8 ± 11.0	55.8 ± 12.8
OD	34.6 ± 14.6 *	35.6 ± 17.2 *
Primary Follicles		
OC	37.4 ± 12.3	33.0 ± 16.2
OD	19.8 ± 5.7 *	21.6 ± 6.4 *
Growing Follicles		
OC	155.8 ± 25.0	148.6 ± 33.6
OD	88.4 ± 16.4 *	89.0 ± 50.5 *
Antral Follicles		
OC	63.4 ± 7.5	50.6 ± 22.4 *
OD	20.0 ± 7.9 *	25.2 ± 12.5 *^#^
Corpus Luteum		
OC	8.8 ± 1.8	7.8 ± 1.6
OD	8.0 ± 1.6	8.2 ± 2.8

Values were expressed as mean ± standard deviation. *n* = 6 rats/group; * *p* < 0.05—compared to the OC/SD group; # *p* < 0.05—compared to the OC/HFD group; (Poisson distribution followed by Wald test—primordial, primary, antral follicles, and corpus luteum; Negative binomial test followed by Wald test—growing follicles).

## Data Availability

Publicly available datasets were analyzed in this study. This data can be found here: http://hdl.handle.net/11449/236737 (accessed on 18 September 2023).
